# Nature Reappraisers, Benefits for the Environment: A Model Linking Cognitive Reappraisal, the “Being Away” Dimension of Restorativeness and Eco-Friendly Behavior

**DOI:** 10.3389/fpsyg.2020.01986

**Published:** 2020-08-06

**Authors:** Angelo Panno, Annalisa Theodorou, Giuseppe Carrus, Claudio Imperatori, Giuseppina Spano, Giovanni Sanesi

**Affiliations:** ^1^Department of Human Science, Cognitive and Clinical Psychology Laboratory, European University of Rome, Rome, Italy; ^2^Department of Education, Experimental Psychology Laboratory, Roma Tre University, Rome, Italy; ^3^Department of Agricultural and Environmental Science, University of Bari, Bari, Italy

**Keywords:** restorativeness, being away, nature, pro-environmental behavior, cognitive reappraisal, emotion regulation

## Abstract

In the last decades, an increasingly prominent role has been given to the motivational factors that can promote pro-environmental behavior. In this contribution, we focus on the role of the individual’s ability to shape the emotions originating from nature in engaging in pro-environmental behavior. In particular, we expect that an emotion regulation strategy as cognitive reappraisal should positively predict pro-environmental behavior, through enhanced perceived restorativeness attributed to the natural environment in terms of the experience of “being away.” One-hundred and fifteen visitors to an urban park (Parco Nord Milano) filled out a questionnaire including measures of cognitive reappraisal, the experience of “being away,” and pro-environmental behavior while in the park. Results confirmed that cognitive reappraisal was positively and significantly related to pro-environmental behavior. Importantly, the indirect effect of cognitive reappraisal on pro-environmental behavior through the experience of “being away” was significant. Findings suggest the importance of implementing interventions aimed at promoting the habitual use of cognitive reappraisal to enhance the experience of “being away” and, thus, sustain pro-environmental behavior.

“We trace out all the veins of the earth, and yet, living upon it, undermined as it is beneath our feet, are astonished that it should occasionally cleave asunder or tremble: as though, forsooth, these signs could be any other than expressions of the indignation felt by our sacred parent!”

– Pliny the Elder

## Introduction

Nature is uncontrollable; however, humankind has a direct impact on some catastrophic natural phenomena. In the last decades, the race for irrepressible productivity resulted in human interference with the course of nature in many essential life processes such as climate change (Crowley, 2000), biodiversity (Hughes et al., 2003), and hydrogeological instability (Vilardo et al., 2009). As Pliny the Elder already claimed in ancient times, we are often ignorant of the catastrophic consequences of human actions and underestimate the enormous importance of the natural environment for human life. In this contribution, we highlight that just as anti-environmental behavior traces back to recklessness and heedlessness, the origins of pro-environmental behavior can be found in the *awareness* of the benefits of nature for the individual’s life.

Awareness, or the ability to be efficiently sensitive to environmental clues, has been related especially to the way individuals regulate their emotions (Gross and John, 2003). Emotions are automatic responses to environmental stimuli, in which individuals may intervene in modulating their intensity and quality (Gross, 1998). Pieces of evidence have shown how a successful management of emotions can boost pro-environmental attitudes and behavior (Aguilar-Luzón et al., 2014; Robinson et al., 2019). In particular, a pioneering study demonstrated how cognitive reappraisal, defined as the tendency to change the way one thinks of a situation to alter the emotional response linked to it, promotes pro-environmental behavior (Panno et al., 2015). Through reappraisal, individuals may elaborate and more deeply understand the emotions elicited from the natural environment, enhancing the likelihood to protect it by engaging in pro-environmental behavior.

In the present research, we intend to shed light on the mechanism between cognitive reappraisal and pro-environmental behavior. In doing so, we will refer to the concept of restorativeness attributed to a natural environment (Hartig and Mang, 1991) namely the individual’s recognition of the beneficial effect of the natural environment. In doing so, we focus on one particular dimension of restorativeness, namely the perception of “being away” experienced in the natural setting. Specifically, we hypothesize that the more the habitual use of the cognitive reappraisal strategy in everyday life, the higher the engagement in pro-environmental behavior. Importantly, the habitual use of reappraisal should promote pro-environmental behavior *through* the enhanced individual perception of restorativeness attributed to the natural environment in terms of the “being away” experience.

### Reappraisers and Pro-environmental Behavior: A Particular Sensitivity

Emotions are important carriers of environmental information (Gross, 1998). Within the realm of emotion regulation strategies particular attention is given to the way individuals “shape” their emotions. Cognitive reappraisal has been defined as a strategy that aims at reconstructing an emotionally charged situation in a way that alters its emotional impact (Gross and John, 2003). As compared to other strategies, cognitive reappraisal has the advantage of being able to intervene on the emotional response to cues before it is fully experienced (Ochsner and Gross, 2008). This early regulation allows more complete control and is more likely to prevent adverse emotional states, as compared to a delayed intervention strategy such as emotional suppression (Wallace-Hadrill and Kamboj, 2016; Dryman and Heimberg, 2018).

The cognitive reappraisal strategy is not a simple concept as it involves at least two mechanisms that correspond to two separate neurological substrates (Buhle et al., 2014). These latter are (a) finding an alternative interpretation of a situation and (b) detaching oneself from a situation that generates an intense emotional state (Ochsner and Gross, 2008). Either way the core element of this strategy is the powerful role of the cognitive reinterpretation of external, but also internal, cues with the specific goal to augment or reduce the emotional charge of circumstances (McRae et al., 2012). Cognitive reappraisal has been demonstrated to sustain psychological well-being by reducing anxiety and depression, and thus resulting in satisfaction with life (Gross and John, 2003; Moore et al., 2008; Hu et al., 2014).

The advantages of cognitive reappraisal are not only related to individual control over emotions. In fact, important results of the adoption of this strategy are extended to the individual’s understanding of the external environment. Emotions are responses to stimuli and individuals who can understand them better are more likely to decode the feedbacks that the environment sends. This mechanism should allow a deeper connection with the environment. In this regard, studies on the way individuals interact with their social environment demonstrated that cognitive reappraisal leads to more positive social relationships than the emotional suppression strategy (Butler et al., 2003; Gross and John, 2003). This would be so because of the enhanced ability to correctly process social information (Gross and John, 2003; Manera et al., 2014) and, by diminishing the ambiguity of the social interaction (Yurtsever, 2008) lead to a better adaptation.

As individuals who habitually use cognitive reappraisal engage in positive prosocial behavior through their efficient reading of the social environment, likewise a correct interpretation of cues originating from natural environments has been demonstrated to lead to higher engagement in pro-environmental behavior (Panno et al., 2015). In other words, we argue that the key role of cognitive reappraisal would be the higher recognition of the personal positive experience related to immersion in the natural environment. Past research showed how awareness of the environmental related issues does not always result in higher engagement in pro-environmental behavior. Indeed, it seems that the role of awareness is effective only when it can stimulate a higher sensitivity in terms of higher environmental related emotions (Hungerford and Volk, 1990; Carmi et al., 2015).

It is well-known that emotions such as guilt and worry constitute an important predictor that indicates whether the individual would engage in pro-environmental behavior (Bamberg and Möser, 2007; Ojala, 2007; Carrus et al., 2008). Importantly, we argue that it is not the emotions *per se*, but the way individuals manage their emotions that impact on the likelihood to adopt pro-environmental behavior. Successful processes of identifying and regulating emotions have been seen as motivational triggers to enhance pro-environmental intentions and behavior (Carmi et al., 2015). For instance, reflecting on worries as a coping strategy reduces this potentially counter-productive state, allowing individuals to learn from the situation. This mechanism, in turn, leads to important pro-environmental behavior (Ojala, 2007). In this regard, we propose that an emotion regulation strategy (i.e., cognitive reappraisal) that allows one to rethink one’s personal experiences gives to the individual a higher awareness of the benefits of nature. This enhanced awareness should lead, in turn, to higher sensitivity in relation to the natural environment. Thus, we expect:

H1: Higher habitual use of cognitive reappraisal will result in higher pro-environmental behavior reported.

### The Experience of Being Away as the Transporter of the Positive Effect of Cognitive Reappraisal on Pro-environmental Behavior

In a relative recent study, it has been shown that cognitive reappraisal promotes pro-environmental behavior through heightened perceptions of climate change (Panno et al., 2015). The basic idea was that the habitual use of cognitive reappraisal makes individuals more sensitive to environmental clues signaling climate change such as, for instance, an increase in the temperatures. This enhanced consideration would transfer to a higher reactivity to these particular cues, consequently traducing in behavior that could reduce the individual footprint on the natural environment. Following the same reasoning, another mechanism through which cognitive reappraisal may relate to pro-environmental behavior is the attention given to the internal cues that originate in response to the natural environment and the subsequent recognition of its value. This particular ability falls within the definition of what has been called restorativeness.

The concept of restorativeness attributed to a natural environment has been defined as both emotional and cognitive responses elicited by contact with nature (Berto, 2014; Martínez-Soto and González-Santos, 2020). In particular, it has been argued that the restorative quality of nature lies in the possibility to suspend direct, and costly, attention, determining an opportunity for cognitive recovery (Kaplan, 1995; Van den Berg et al., 2007). A particular process involved in restorativeness is what has been called the experience of “being away” (Kaplan and Kaplan, 1989; Hartig et al., 1996). This perception is defined as the feeling of psychological distance from daily demanding routines. It comprises the feeling of avoiding environmental distractions, of escaping from one’s routine, and of suspending the possibly costly process of pursuing specific purposes (Hartig et al., 1996).

Restorativeness has been positively related to indicators of physical and psychological health (Hartig et al., 2014; Carrus et al., 2015; McMahan and Estes, 2015). In this regard, past research demonstrated how some individual differences can either diminish or enhance the perceived restorativeness of a natural environment (Korpela et al., 2001; Scopelliti et al., 2016; Berto et al., 2018). For instance, the habitual use of specific emotion regulation strategies or even personality traits may intervene in determining perceived restorativeness (Korpela et al., 2001, 2008; Johnsen, 2013). Restorativeness, as a quality attributed to a natural environment, is related to the individual capacity to fully understand the potentiality of exposure to nature (Carrus et al., 2017). In particular, individuals who feel more connected with nature report higher levels of restorativeness (Berto et al., 2018; Mena-García et al., 2020).

There is reason to hypothesize that individuals who exhibit higher habitual use of cognitive reappraisal would engage more in the process of elaborating and recognizing the utility of the natural environment, meaning its value for one’s personal restorativeness. In our reasoning, we focus particularly on the dimension of “being away.” As it has been argued, this dimension is related to the individual recognition of the utility of the natural setting as a way to “escape” from costly distractions and demands coming from one’s daily routines (Staats, 2012). In this regard, we believe that, among others, is this dimension that is the most related to the individual ability to be aware of the importance and value of the natural environment. Therefore, individuals who report higher habitual use of cognitive reappraisal should show a higher appreciation of the natural potentiality to restore in terms of “being away” from duties and demands. Accordingly:

H2: Higher habitual use of cognitive reappraisal strategy will lead to higher experience of “being away” attributed to the natural environment.

Studies demonstrated that higher perceived restorativeness attributed to the natural environment is associated with higher pro-environmental beliefs and behavior (Hartig et al., 2001; Collado and Corraliza, 2015, 2017). It has been argued that restorativeness may function as a motivational factor that could determine the choice to adopt a positive behavior toward the environment (Berto and Barbiero, 2017). Indeed, embracing the utility of nature should motivate individuals to engage in behaviors that could protect it. Thus, our third prediction is that the more the perceived utility of nature in terms of the “being away” experience, the higher the engagement in pro-environmental behavior:

H3: Higher perceived experience of “being away” will predict higher pro-environmental behavior reported.

In sum, in line with previous mediational studies on the topic (Van den Berg et al., 2003; Johnsen, 2013) the purpose of the present study is to test a mediational model. The hypothesis is that cognitive reappraisal positively predicts pro-environmental behavior through the awareness of the utility of the natural environment for one’s life in reference to the “being away” experience. The rationale is that, through their cognitive reappraisal, individuals have the opportunity to better understand the positive cues prompted by the natural environment and feel more connected with it. Thus, they would appreciate nature more and feel more restored by exposure to nature. This sense of restoration would then augment the relative importance of defending the environment, leading to more pro-environmental behavior. Therefore, our fourth and last prediction is:

H4: Cognitive reappraisal positively predicts the engagement in pro-environmental behavior through the enhanced experience of “being away.”

**FIGURE 1:**
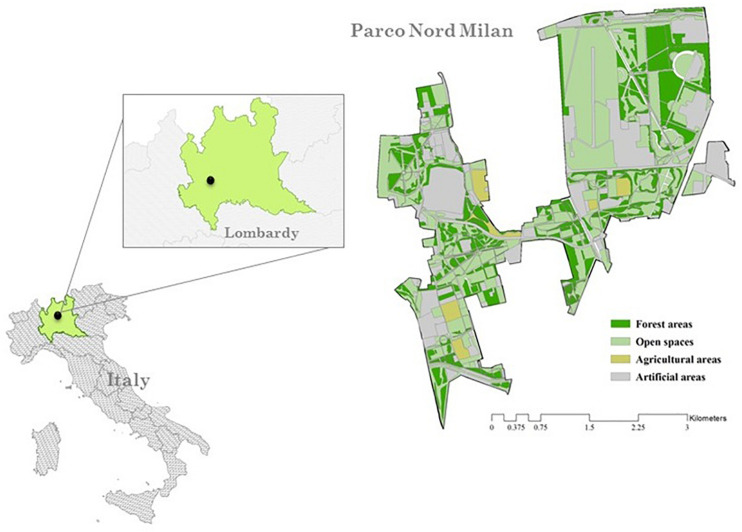
Location of the study area – Parco Nord Milan, Italy.

Following the importance of parks for individual restoration (Peschardt and Stigsdotter, 2013) we tested our hypotheses on a sample of visitors to Parco Nord Milano (PNM). PNM is a public park located in the north-east side of the metropolitan area of Milan (see Figure 1). The park covers more than 600 ha; about 100 ha are forests; the remaining part is covered by fields (211 ha), social allotments (2.1 ha), hedges (0.84 ha), rows of trees (14.4 ha), recreational facilities, small lakes, gray infrastructures (schools, hospital, private airport) and agricultural areas. PNM is an important forestation started since 1983. PNM represents a specific type of Nature Based Solution (NBS) (European Commission, 2015) and consists of reclaimed post-industrial or uncultivated lands (Sanesi et al., 2007).^1^ We asked participants to report their perceived experience of “being away” attributed to the park and collected measures of cognitive reappraisal and pro-environmental behavior.

## Materials and Methods

### Participants

One-hundred and fifteen participants visiting PNM participated in the study on a voluntary basis. To be confident in the soundness of the results that we found, we then performed a *post hoc* power analysis using the R application written by Schoemann et al. (2017). In line with a previous study (Panno et al., 2015) detecting a small-medium correlation between cognitive reappraisal (IV) and pro-environmental behavior (DV), we then set small-medium correlations between the independent and the dependent variable (i.e., 0.25) and between the independent variable and the mediator (being away) (i.e., 0.30). We also set a large correlation (i.e., 0.40) between the mediator and the dependent variable. Moreover, we chose values of 5,000 for the total number of power analysis replications and values of 20,000 for the number of coefficients draws per replication (Schoemann et al., 2017). The analysis revealed a statistical power of 0.88 with a sample size of 115 participants.

The sample covered a wide age range (19–81 years). The mean age of the sample was 43.27 years (SD = 16.96). The gender composition was also balanced (49% women). The education level of participants varied from primary school to university degree as follows: 10% primary school and secondary school, 38% high school, 50% university degree. They reported their employment status as follows: 52% intellectual work, 10% manual work, 10% student, 18% retired, and 4% unemployed. The household composition of the sample was as follows: 47% stated living along with partner and children, 21% stated living along with partner but without children, 9% stated having a partner (not living together) and 15% of the sample was single. Research data are available on request without restrictions.

### Procedure and Measures

Data were collected through a paper and pencil questionnaire that included variables concerning socio-demographics, as well as psychological constructs. The questionnaire was administered by trained research assistants who approached participants at PNM, asking them to voluntarily take part in a survey on issues related to green spaces and well-being. Participants individually filled in the questionnaire in a time limit of about 15 min, and were not given any financial compensation. Individuals were asked to answer to the questionnaire basis on their everyday experience, while for the “being away” experience individuals were given specific instructions to refer to their experience while in PNM. The questionnaires were collected in September 2015. They were assured anonymity about their responses. All procedures performed in studies involving human participants were conducted in accordance with the ethical standards of the institutional and national research committee and the 1964 Declaration of Helsinki and its later amendments or comparable ethical standards. The article does not refer to any studies with animals performed by any of the authors.

Because this study was part of a larger survey aimed at investigating other factors unrelated to the aims of the present study (e.g., perception of an increase in the temperatures), we then measured some constructs with the sub-scale reflecting the dimension of our interest (e.g., cognitive reappraisal of emotion regulation questionnaire) and an abbreviated measure of pro-environmental behavior. We assessed people’s cognitive reappraisal strategy by using the cognitive reappraisal dimension of the short Italian version of the Emotion Regulation Questionnaire (it is composed of four items; Balzarotti, 2019). Respondents rated the extent to which they agree with self-descriptive statements reflecting cognitive reappraisal. An example of item: “*When I want to feel more positive emotion, I change the way I’m thinking about the situation*.” Ratings were made on a 5-point Likert type scale, with the response anchored at the ends with 1 (strongly disagree) and 5 (strongly agree) (*M* = 2.92, SD = 0.72; α = 0.8 and ω = 0.8; Dunn et al., 2014; see also Hayes and Coutts, 2020 for more details).

The being away dimension of the Perceived Restorativeness measure was assessed through all of three items of this sub-scale of the Perceived Restorativeness Scale – Short Version (Scopelliti et al., 2012; Pasini et al., 2009). The short version of the PRS scale has been validated in Italian and English. The being away sub-scale is one of the four PRS dimensions reported by Pasini et al. (2009). An example of item: “*To get away from things that usually demand my attention I like to go to places like this*” Ratings were made on a 5-point Likert type scale, with the response anchored at the ends with 1 (strongly disagree) and 5 (strongly agree) (*M* = 3.38, SD = 0.79; α = 0.7 and ω = 0.7).

Due to the time limit to administer the questionnaire, only one restorative dimension has been assessed. The wording of being away items explicitly links the park’s restorative qualities to benefits for people (e.g., *to stop thinking about the things that I must get done I like to go to places like this* – being away item). In other words, it is a PRS dimension focusing on the properties of the park in giving rise to cognitive restoration (i.e., the benefits for people); whereas other dimensions, such as coherence, concern mainly the physical qualities of the park and the link of such qualities with the benefits for individuals is not explicit in the wording of the items (e.g., *There is a clear order in the physical arrangement of places like this* – coherence item). Thus, the “being away” dimension better captures the link between the restorative quality of the park and benefits for people.

To assess pro-environmental behavior, we used five items measuring people’s tendency to engage in eco-friendly behavior. Some instances of item are: “*Recycle paper, plastic, and metal*,” “*Avoid using public transportation*” (reverse score), “*Replace incandescent light bulbs with CFLs*” (Panno et al., 2018). A composite score of these items indicated environmentally responsible behavior that people adopt in order to reduce their ecological footprint. Ratings were made on a 5-point Likert type scale, with the response anchored at the ends with 1 (strongly disagree) and 5 (strongly agree) (*M* = 3.26, SD = 0.59; α = 0.6 and ω = 0.6.

### Statistical Analysis

**FIGURE 2:**
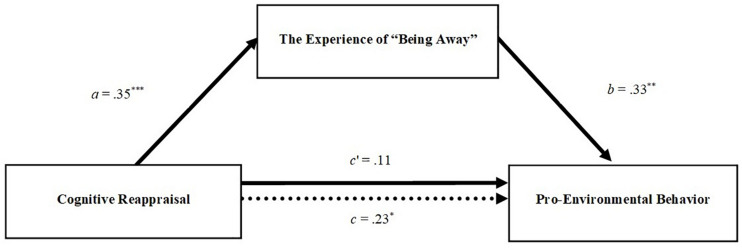
Path coefficients for mediation analysis in the study. Dotted line denotes the effect of cognitive reappraisal strategy on pro-environmental behavior, when the experience of “being away” is not included as a mediator. a, b, c, and c′ are standardized OLS regression coefficients. *p < 0.05; **p < 0.01; ***p < 0.001.

To investigate the relationships among cognitive reappraisal, being away, and pro-environmental behavior, we firstly computed the zero-order correlations among these and socio-demographic variables. We then used the PROCESS macro (Hayes, 2012) which allowed us to test the role of being away as a mediator of the relationship between cognitive reappraisal and pro-environmental behavior. This macro tested the steps of mediation (see also Baron and Kenny, 1986). First, we tested the total effect of cognitive reappraisal on pro-environmental behavior (step 1). In the second step, we tested the effect of cognitive reappraisal on being away, meaning the effect of the predictor on the mediator (path *a –* see Figure 2). In the third model (step 3 and 4), we posited cognitive reappraisal and being away as predictors of pro-environmental behavior, testing simultaneously both effects of the mediator on the outcome (path *b –* see Figure 2) and the direct effect of the predictor on the outcome (path *c′–* see Figure 2). Lastly, we tested the significance of the indirect effect.

We included socio-demographic variables (i.e., gender, age, household composition, education level, employment status) in the mediation analysis to control for potential confounding variables in the results of the study. A bootstrapping procedure (with 5,000 bootstrap samples) to estimate 95% confidence intervals (95% CI) was used. According to Preacher and Hayes (2008) a 95% CI that does not include zero provides evidence of a significant indirect effect. The bootstrapping procedure has been suggested to represent the most trustworthy test for assessing the effects of mediating models (Hayes and Scharkow, 2013 for a recent review). The 0.05 level of significance was adopted throughout all analyses.

## Results

As shown in Table 1, cognitive reappraisal was positively and significantly correlated with pro-environmental behavior. This latter result supports the positive total effect of cognitive reappraisal on pro-environmental behavior hypothesized in H1 (step 1). In addition, the experience of “being away” was also positively and significantly related to pro-environmental behavior. Our results also showed that cognitive reappraisal was significantly and positively correlated with the experience of “being away.” The effect sizes of these relationships were around the moderate effect size threshold as they ranged from *r* = 0.24 to *r* = 0.36 (Cohen, 1988; see Table 1).

**TABLE 1:** Means, standard deviations, and intercorrelations among variables investigated in the study.

	1	2	3	4	5	6	7	8
1. Cognitive reappraisal	1							
2. Being away	0.35***	1						
3. Pro-environmental behavior	0.24*	0.36***	1					
4. Age	–0.02	–0.12	0.09	1				
5. Gender	0.01	0.25**	0.04	–0.18	1			
6. Education	0.17	0.06	0.18	−0.34***	0.14	1		
7. Household composition	0.16	0.26**	0.07	−0.47***	0.04	0.11	1	
8. Employment status	0.05	0.03	–0.08	−0.45***	0.14	0.53***	0.06	1

*p < 0.05; **p < 0.01; ***p < 0.001.

To understand the mechanisms underlying the relationships between cognitive reappraisal strategy, the experience of “being away,” and pro-environmental behavior, we tested the mediation hypothesis. The mediation model was estimated to derive the total, direct, and indirect associations of cognitive reappraisal strategy with pro-environmental behavior through the experience of “being away.” We estimated the indirect effect of cognitive reappraisal on pro-environmental behavior, quantified as the product of the OLS regression coefficient estimating the experience of “being away” from cognitive reappraisal strategy (path *a* in Figure 2 – step 2), and the OLS regression coefficient estimating pro-environmental behavior from the experience of “being away,” controlling for cognitive reappraisal (path *b* in Figure 2 – step 3 and path *c*′ in Figure 2 – step 4). Overall, these results revealed that both paths *a* and *b* were significant. Indeed, cognitive reappraisal positively predicted the experience of “being away” confirming H2. In turn, controlling for cognitive reappraisal, the experience of “being away” positively predicted pro-environmental behavior confirming H3.

As the last step, we proceeded to test the indirect effect of cognitive reappraisal on pro-environmental behavior. In this regard, a bias-corrected bootstrap-confidence interval (CI) for the product of paths *a* and *b* that does not include zero provides evidence of a significant indirect effect (Preacher and Hayes, 2008). Using the PROCESS macro with 5,000 bootstrap samples, our results revealed a significant positive indirect effect of cognitive reappraisal on pro-environmental behavior through the experience of “being away” (point estimate = 0.113; 95% CI = 0.023–0.226). This latter finding confirmed the hypothesized indirect effect that cognitive reappraisal shows on pro-environmental behavior through enhanced experience of “being away” reported in H4.

Since participants’ gender and age, as well as other socio-demographic variables could be related to both the experience of “being away” and pro-environmental behavior, we also tested a mediating model which included gender (men coded as 1 and women coded as 2), age, education, household composition, and employment status as covariates. The relationships between cognitive reappraisal, the experience of “being away,” and pro-environmental behavior did not substantially change after controlling for the effect of all these covariates (point estimate = 0.118; 95% CI = 0.011–0.260). Interestingly, we found a significant positive effect of gender on the experience of “being away” (β = 0.24, *p* < 0.05), with women perceiving more experience of “being away” than men. We also found a significant effect of age and education on pro-environmental behavior with older (β = 0.30, *p* < 0.05) and well-educated (β = 0.32, *p* < 0.05) people being more environmentally oriented.

## Discussion

In recent years, the negative impact of anthropic activity on the natural environment has been becoming more and more obvious (Spano et al., 2020). In light of the changes in several natural processes we experience nowadays, the importance of shedding light on the antecedent of pro-environmental behavior represents a current challenge (Gifford and Nilsson, 2014). In this regard, we claimed that an important factor that can motivate people to engage in pro-environmental behavior is the awareness of the positive benefits of nature. Specifically, we proposed a mediational model. We hypothesized that using an emotional regulation strategy as cognitive appraisal, individuals may be more in tune with the natural environment and, as a consequence, engage more in pro-environmental behavior. Indeed, through cognitive reappraisal, individuals may appreciate more the value of the time spent surrounded by nature, as indicated by a higher perceived experience of “being away.” This latter, in turn, should enhance engagement in pro-environmental behavior. The overarching message of this work is that cognitive reappraisal (i.e., a coping strategy) copes with the need to shift the attention from daily routine demanding cognitive efforts to the potentially restorative environment (Kaplan, 1995). This rationale is in line with a coping strategy that may bring the individual to use the park as a means to regulate distress arising through daily routine (e.g., things that usually demand attention; Ochsner and Gross, 2008; Buhle et al., 2014).

Our first hypothesis stated that the higher the habitual use of cognitive reappraisal, the higher the pro-environmental behavior reported. This hypothesis was confirmed by the data. Indeed, in line with previous findings (Panno et al., 2015) there was a positive association between the two variables. Cognitive reappraisal intervenes in the way individuals adapt to their environment (Keltner and Gross, 1999). Having the possibility to rationally shape the emotions seems to lead the way to higher importance attributed to the engagement in pro-environmental behavior. This result attests that the well-known benefits of habitual use of cognitive reappraisal in individual psychological and social adaptation may extend to adaptation to the natural environment.

Our second hypothesis was that the more the habitual use of cognitive reappraisal, the more the perceived restorativeness attributed to the natural environment in terms of the experience of “being away.” This hypothesis was confirmed by the data. Indeed, it seems that a greater understanding of the benefits of the environment, achieved through the cognitive reappraisal strategy, sustains the experience of escaping from costly routines and finding “restorativeness” in the natural park. Our third hypothesis was that the more the experience of “being away” attributed to the natural environment, the more the pro-environmental behavior reported. This prediction was confirmed by the data as well. Therefore, a positive experience of stay in the park related with the importance given to the behavioral choices that could protect the natural environment.

Lastly, our fourth hypothesis was that cognitive reappraisal exerts its role in sustaining pro-environmental behavior through an enhanced experience of “being away.” This latter hypothesis was confirmed by a significant indirect effect; importantly, when considering the experience of “being away,” the effect of cognitive reappraisal disappeared, suggesting that the totality of the effect of cognitive reappraisal on pro-environmental behavior passes through the experience of “being away.” These latter findings constitute the most innovative contribution of this research. In fact, as the role of cognitive reappraisal in pro-environmental behavior is just beginning to be studied, it is important to shed light on the mechanism through which it can sustain them. In this regard, it seems that the experience of “being away” plays a central role. Processing social information more effectively results in better social functioning (Gross and John, 2003). Likewise, it seems that the way individuals elaborate cues originating from nature enhances their engagement in pro-environmental behavior: a sort of “natural” functioning. Ultimately, engaging in this behavior is likely to benefit the individual him or herself (Suárez-Varela et al., 2016; Schmitt et al., 2018). Moreover, some socio-demographic variables showed a significant effect on the experience of “being away” as well as pro-environmental behavior. Indeed, these results pointed out that women perceived more benefits, in terms of the experience of “being away,” than men. Finally, older and well-educated people would seem to be more environmentally oriented. Thus, these findings mark again the role of education in the promotion of environmentalism and the relevance of the relationship between elderly and younger people in the stimulation of a pro-environmental stance in society at large.

This research is not free of limitations. First of all, the cross-sectional nature of the research makes it impossible to ascertain causal relationships. Thus, future studies with experimental or longitudinal designs are needed in order to test causation. Moreover, cross-sectional designs could give rise to common method variance issue. Thus, although such an issue should not undermine novel research avenues, future studies should investigate these relationships through experimental designs. Secondly, the peculiar features of PNM may limit the generalization of our model to other urban parks and other natural areas. In particular, the emotional response to the natural setting offered by an urban park may differ from the experience of wilder natural settings. Thus, future studies can test our model with reference to wilder natural areas, such as forests. Due to the narrowed time limit that we had to administer the questionnaire, in the current study we sought to assess the constructs of interest through some dimensions of the respective scale (e.g., we only assessed the cognitive reappraisal dimension of the emotion regulation questionnaire). Clearly, these results provide a first step in this avenue of research and future studies using the full scale of the constructs may add robustness to these findings. For example, future studies should use the full version of the PRS scale.

From a theoretical point of view, it has been noted that urban parks can cause ambiguous feelings in the city’s inhabitants (Burgess et al., 1988). During the stay in urban parks, positive feelings may be accompanied by negative feelings (e.g., fear for one’s own safety, perception of low control on the environment, and reduction of personal comfort; Bonnes et al., 2011). The conjunction of negative and positive emotions is important for engaging in pro-environmental behavior (Ojala, 2007). It is possible that both explicit and implicit mechanisms are involved in this process, as suggested by previous studies in different domains, such as esthetic preference (e.g., Mastandrea et al., 2011; Mastandrea and Maricchiolo, 2014). Thus, future research could address the specific role of cognitive reappraisal on both positive and negative feelings originating from the park experience and their relationship with pro-environmental behavior. The present work might have relevant applied implications. For instance, future research could explore the possibility of designing cognitive reappraisal trainings (Denny and Ochsner, 2014) through natural settings. Such a line of research could shed light on common motivational factors underlying the relationship between perceived natural restorativeness in terms of the experience of “being away” and pro-environmental behavior. Moreover, such interventions might be adopted in school settings to promote an environmentalist attitude among the young.

## Conclusion

In conclusion, understanding the way individuals “sense” their environment is important in order to predict their behavior. A deeper understanding of this mechanism is essential in order to design increasingly sophisticated interventions aimed at enhancing pro-environmental behavior. First and foremost, particular attention needs to be directed towards those factors that can be sustained and promoted through training. This study’s results point to an emotion regulation strategy as cognitive reappraisal as a possible target of psychological intervention. If future studies could confirm the study findings, these latter could have an important implication in interventions aimed at sustaining pro-environmental choices. In fact, as an emotion regulation strategy, the habitual use of cognitive reappraisal is susceptible to change: it can be sustained and taught to promote pro-environmental behavior.

## Data Availability Statement

The raw data supporting the conclusions of this article will be made available by the authors, without undue reservation.

## Ethics Statement

All procedures performed in studies involving human participants were conducted in accordance with the ethical standards of the institutional and national research committee and the 1964 Declaration of Helsinki and its later amendments or comparable ethical standards. Written informed consent for participation was not required for this study in accordance with the national legislation and the institutional requirements.

## Author Contributions

AP: conceptualization. AP and CI: methodology. AP, GSp, and CI: statistical analysis. GSp: data acquisition. AT and AP: writing–first draft and final draft of the manuscript. GC and GSa: review and editing. All authors contributed to the article and approved the submitted version.

## Conflict of Interest

The authors declare that the research was conducted in the absence of any commercial or financial relationships that could be construed as a potential conflict of interest.

## Footnotes

1 http://parconord.milano.it/
